# Encapsulation of *Saccharomyces pastorianus* Residual Biomass in Calcium Alginate Matrix with Insights in Ethacridine Lactate Biosorption

**DOI:** 10.3390/polym14010170

**Published:** 2022-01-01

**Authors:** Lăcrămioara Rusu, Cristina-Gabriela Grigoraș, Andrei-Ionuț Simion, Elena-Mirela Suceveanu, Alexandra-Cristina Blaga, Maria Harja

**Affiliations:** 1 Faculty of Engineering, “Vasile Alecsandri” University of Bacau, 157 Calea Mărăşeşti, 600115 Bacau, Romania; asimion@ub.ro (A.-I.S.); mirela.suceveanu@ub.ro (E.-M.S.); 2 Faculty of Chemical Engineering and Environmental Protection “Cristofor Simionescu”, “Gheorghe Asachi” Technical University from Iasi, 71 A Mangeron Blvd., 700050 Iasi, Romania; acblaga@tuiasi.ro

**Keywords:** encapsulation, natural polymer, pharmaceuticals, yeast biomass, water treatment

## Abstract

Pharmaceuticals are recognized as emerging water microcontaminants that have been reported in several aquatic environments worldwide; therefore, the elimination of these pollutants is a global challenge. This study aimed to develop a biosorbent based on *Saccharomyces pastorianus* residual biomass encapsulated in a calcium alginate matrix and to evaluate its biosorption performance to remove Ethacridine Lactate (EL) from aqueous solutions. Firstly, the synthesis and characterization of biosorbent has been carried out. Then, the impact of main parameters on biosorption process were investigated by batch experiments. Finally, the kinetics behavior and equilibrium isotherms were evaluated. The resulted beads have an irregular and elongated shape with about 1.89 mm ± 0.13 mm in size with a homogeneous structure. The best removal efficiency for EL of over 85% was obtained at acidic pH 2 and 25 °C for 50 mg/L initial concentration and 2 g/L biosorbent dose. The pseudo-second-order and intraparticle diffusion kinetics describe the biosorption process. The maximum calculated biosorption capacity was 21.39 mg/g similar to that recorded experimentally. The equilibrium biosorption data were a good fit for Freundlich and Dubinin–Radushkevich isotherms. Our findings reveal that the low cost and eco-friendly obtained biosorbent can be easily synthesized and suitable to remove Ethacridine Lactate from water matrices.

## 1. Introduction

The presence of persistent organic pollutants, considered as emerging contaminants, in different environmental matrices is a major concern worldwide [1,2]. Water resources are increasingly becoming limited, and quality of water bodies has been seriously threatened by the presence of different contaminants that pose a risk to the human health and the aquatic environments. Water resources are increasingly becoming limited, and quality of water bodies has been seriously threatened by the presence of different contaminants that pose a risk to the human health and the aquatic environments [3]. Of current major concern are emerging organic micropollutants such as pharmaceuticals and personal care products [4,5,6,7,8].

In the last two decades, pharmaceuticals were detected in surface waters, seawaters, groundwater, drinking water and sewage treatment plants effluents [9,10,11,12]. Reported concentrations of these compounds in surface water are between 6.46 ng/L and 9822 ng/L [1].

To avoid the negative impact of pharmaceuticals it is necessary to develop technology for their complete removal from wastewater before they are discharged into environment [1]. Previous methods investigated to this purpose include: coagulation, adsorption, ion exchange, advanced oxidation, photocatalysis, sol-vent extraction, membrane separations and biological degradation [2,6,7,10,13,14,15,16]. These procedures have different advantages and disadvantages which involves efficiency, sustainability and cost. Conventional wastewater treatment methods fail to eliminate pharmaceuticals totally from the water. However, the adsorption process remains relevant due to its simplicity, easy maintenance, good efficiency and low cost [1,17,18,19,20,21].

Biosorption using natural polymers as support for biomass can represent an alternative to these methods if efficient, cheap, non-toxic and easily available biosorbents can be found or synthesized [22].

The ability of microorganisms (i.e., bacteria, fungi, algae) and microbial residual bio-mass for removal of pharmaceuticals from aqueous solution have been studied. Even if in most cases a good removal efficiency was obtained, their applications in wastewater treatment are limited due to difficulties in their separation from effluents that involves an increase of cost. 

The free cells encapsulation or immobilization in a fixed matrix constitute a solution to this problem, offering at the same time a series of advantages such as: easy solid–liquid separation, control of the size, design of different type of biosorbents and possibility of use in continuous flow systems [17,18,23,24,25,26].

The encapsulation method was first investigated for applications in the food and pharmaceutical industries, then it was extended to wastewater and waste stream treatment [25]. The natural and synthetic polymers and inorganic materials have been studied as matrices in encapsulation technology [25,27,28]. 

Among the natural polymers, the most used for biosorbents obtaining is the alginate due to its good chemical stability, non-toxicity, biocompatibility, incorporation efficiency and low cost [24,29].

By the encapsulation technology, one can obtain capsules with different shapes (sfer-ic, irregular), sizes (nanocapsules, microcapsule, millicapsule) and morphologies which depend of preparation techniques: chemical methods (i.e., interfacial, in situ polymerization), physicochemical methods (i.e., coacervation, sol-gel, layer-by-layer), and physicome-chanical (i.e., spray-drying, co-extrusion, phase-inversion precipitation) [27].

Therefore, the encapsulation of microbial biomass in a biopolymer matrix can lead to an efficient, much more stable, economical and safe biosorption system [20]. 

Among the most extensively researched microorganisms are the yeasts from *Saccha-romyces* genus, especially *Saccharomyces cerevisiae*, which have been noted as efficient biosorbents for heavy metals, dyes and drugs [17,18,19,20,23,24,29,30].

*Saccharomyces pastorianus* is a cool bottom-fermenting yeast currently used in the brewing industry [18]. The residual biomass of *Saccharomyces pastorianus* is a by-product generated by this process which is available in large quantities if take into consideration the global beer production of 1.82 billion hectoliters in 2020 [31]. Even though this biomass is safe and available, only few studies reported its use in biosorption processes [18,29]. Therefore, this can be both a feasible option and a novelty in the biosorbents field.

Ethacridine lactate (EL) (6,9-diamino-2-ethoxyacridine lactate monohydrate) is an aromatic organic compound based on acridine. In solutions of 0.1%, this cationic drug is frequently used as an antiseptic [2]. Further, it has various uses in both human and veterinary medicine, for example, as a potent antimicrobial agent in the treatment of human chronic diarrhea [32], bovine streptomastitis [33] or in ear-drop formulations [2]. 

Although, Ethacridine lactate is a drug used worldwide in large quantities, to date, only few studies have been reported regarding its removal from water. For this purpose, adsorption on activated carbon and bentonite was used [2,34].

To the best of our knowledge, the possibility of removing EL from water using micro-organisms and residual biomass was first mentioned in our previous study [29]. 

The obtained results for six types of biosorbents prepared by immobilization highlighted the fact that research can be continued in order to develop mathematical models and to optimize the biosorption process and can be extended by using encapsulation method for biosorbents synthesis.

In this context, the actual study aimed to develop a biosorbent based on *Saccharomyces pastorianus* residual biomass encapsulated in a calcium alginate matrix and to evaluate its biosorption performance to remove EL from aqueous solutions. 

Our work was focused on the synthesis and characterization of biosorbent and also on the equilibrium and kinetics approaches of biosorption process. 

The proposed strategy provides an eco-friendly drugs removal system with potential applications in different water matrices when conventional methods are not feasible.

## 2. Materials and Methods

### 2.1. Reagents and Analytical Procedure

All reagents necessary for the present research work were of analytical purity and were used without prior treatment or purification. 

**Figure 1:**
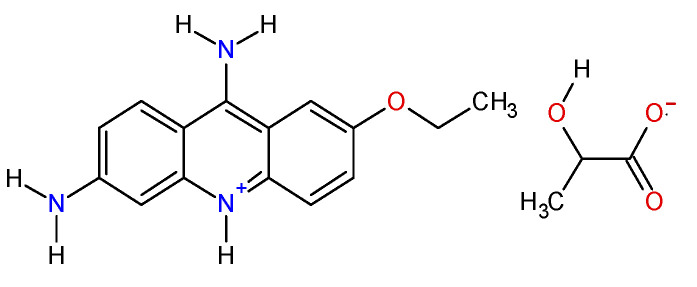
Ethacridine lactate (EL) structure (CAS 1837-57-6; molecular formula: C_18_H_21_N_3_O_4_; MW = 343.4 g/mol).

Ethacridine lactate (Figure 1) was bought from Merck (Darmstadt, Germany). Hydrochloride acid, sodium chloride and ethanol were provided by Chemical Company (Iași, Romania). Sodium hydroxide and calcium chloride were purchased from Chempur (Piekary Ślaskie, Poland). Sodium alginate (low viscosity grade) was procured from BUCHI Laboratortechnik AG (Flawil, Switzerland).

Residual biomass of *Saccharomyces pastorianus* was kindly donated by Albrau Company (Onești, Romania). 

All the solutions were prepared in distilled water. When needed, NaOH (0.1 M) or HCl (0.1 M) were used to adjust the pH.

From a 500 mg/L stock solution of Ethacridine lactate, kept at 4 °C in a closed vessel, dilutions within concentration range from 1 mg/L to 70 mg/L were obtained. Their absorbance was read at 431 nm on a UV1280 spectrophotometer (Shimadzu, Tokyo, Japan) and served for plotting the calibration curve. 

All the experiments were carried out in triplicate. 

### 2.2. Biosorbent Synthesis and Characterization

In a solution of sodium alginate (2% *w*/*w*), the adequate amount of residual biomass of *Saccharomyces pastorianus* was added in order to attain a final concentration of 5%. After proper homogenization, by dripping method, the suspension was passed through a Buchi encapsulator B-390 (Buchi Laboratortechnik AG, Flawil, Switzerland) whose specific parameters were set as follows: vibration frequency, 400 Hz; electrode tension, 900 V; temperature, 45 °C; into a 2% calcium chloride solution. The formed capsules were then washed with distilled water and kept in calcium chloride solution for at least 24 h. Before being used in sorption experiments, they were washed again to insure the complete removal of the storage solution. 

The obtained biosorbent was characterized by Scanning Electron Microscopy (SEM), Fourier Transform Infrared Spectroscopy (FTIR) and by establishing its point of zero charge (pH_PZC_). 

In the first case, a SEM Quanta 200 3D (FEI Europe B.V., Eindhoven, The Netherlands) apparatus equipped with an energy-dispersive X-ray system was employed. The capsules were dried at 50 °C for 2 h in an Air Performance AP60 hot air oven, (Froilabo, Paris, France) and then fixed to stubs with double adhesive carbon discs. Normal secondary electron mode (SE) in low vacuum was applied while the detection was ensured by a large field detector (LFD) at an accelerating voltage of 20 kV, a working distance of 14.6–15.5 mm and a spot size of 5. The magnification range was between 1 mm and 10 μm. 

In the second case, FTIR spectra were recorded from 4000 cm^−1^ to 400 cm^−1^ (32 sample/background scans) with a spectral resolution of 4 cm^−1^ on a Nicolet iS50 FTIR spectrometer (Thermo Scientific, Dreiech, Germany) coupled with a built-in ATR accessory. The ATR cleaning was performed with ethanol after each spectrum registration. The background spectrum reference with air was recorded and compared with the anterior one. 

For pH_PZC_ determination, 0.4 g of biosorbent were placed in laboratory beakers. Aliquots of 20 mL of 0.1 M NaCl solutions with initial pH values (pH_i_) between 2 and 12 measured with a portable pH meter (Dostmann KLH9.1, 0–14 pH, Carl Roth, Karlsruhe, Germany) were added. The mixtures were agitated for 24 h on magnetic plates at ambient temperature. Then, the pH values (pH_f_) were measured again and graphically represented against pH_i_.

### 2.3. Influence of Biosorption Operational Parameters (pH, Adsorbent Amount, Initial Pollutant Concentration, Working Temperature)

A series of experiments were carried at initial pH of EL solutions (60 mg/L) between 2 and 10 with 1 g/L of biosorbent beads. The biosorbent concentration was then varied from 1 g/L to 3 g/L. After that, different EL initial concentrations (20 mg/L to 60 mg/L) were tested. Finally, the effect of working temperature was studied. The contact period for the determinations was set at 24 h.

The residual EL concentrations were calculated by reading the samples absorbance at 431 nm against the calibration curve.

The removal efficiency (R, %) and the biosorption capacity at equilibrium (*q_e_*, mg/g) were calculated with the subsequent equations:

(1) $$R=\frac{\left(C_{0}-C_{e}\right)}{C_{0}}\cdot 100$$

(2) $$q_{e}=\frac{\left(C_{0}-C_{e}\right)\cdot V}{m}$$

in which *C*_0_ and *C_e_* are the initial and at equilibrium EL concentrations (mg/L), *m* is the biosorbent concentration (g/L) and *V* is the volume of EL used for the experiments (L).

### 2.4. Kinetics and Equilibrium Isotherms

Several kinetic models such as pseudo-first-order, pseudo-second-order, and intraparticle diffusion were tested and different equilibrium isotherms, i.e., Langmuir, Freundlich, Dubinin–Radushkevich and Temkin were used to validate the uptake behavior of Ethacridine lactate by the biosorbent obtained by encapsulation of *Saccharomyces pastorianus* residual biomass on calcium alginate.

## 3. Results and Discussion

### 3.1. Biosorbent Preparation 

**Figure 2:**
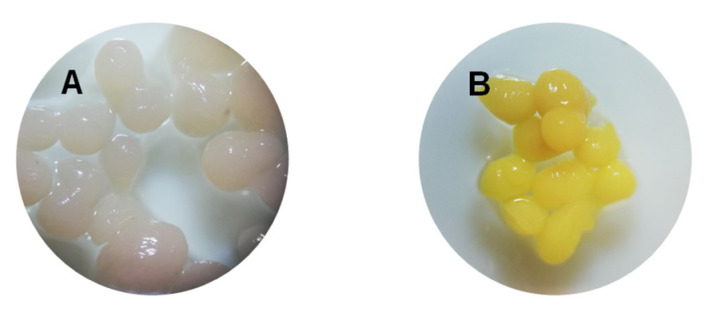
Photographs of the synthesized biosorbent before adsorption (**A**) and after adsorption (**B**) of Ethacridine lactate from aqueous solution.

Sodium alginate possess exceptional gelling properties and has the ability to stimulate the cross-linking polymerization and the formation of an elastic environment [35]. Thus, this natural polymeric matrix is considered suitable for the entrapment of the residual biomass of *Saccharomyces pastorianus*. The viscous suspension obtained by mixing sodium alginate with biomass was forced to pass through the encapsulator small diameter nozzles under high pressure. As consequence, the resulted beads (Figure 2) have an irregular and elongated shape and not the spherical and regular one attained, for example, when the immobilization procedure is applied [24]. The beads dimensions were between 1.89 mm ± 0.13 mm. 

### 3.2. Biosorbent Characterization (SEM, FTIR, and Point of Zero Charge)

**Figure 3:**
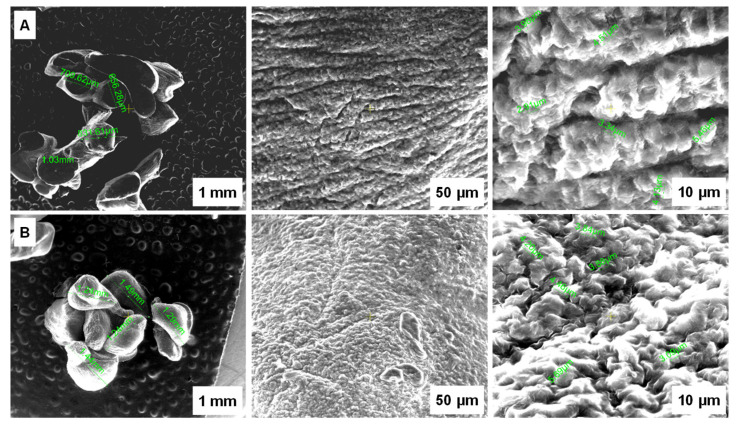
SEM images of the synthesized biosorbent before adsorption (**A**) and after adsorption (**B**) of ethacridine lactate from aqueous solution.

SEM analysis of the dried capsules is depicted in Figure 3. A homogeneous structure with rolling tendencies can be observed before adsorption of EL from aqueous media while a smoother surface and a uniform pore distribution can be noticed after the process completion. Changes of beads morphology sustain the fact that the target drug was retained by the prepared biosorbent. 

**Figure 4:**
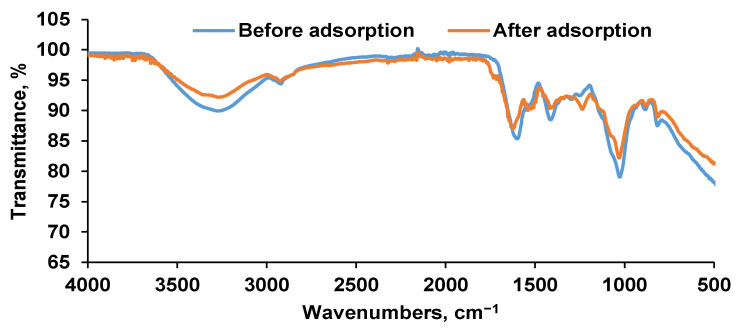
FTIR spectra of the synthesized biosorbent before adsorption and after adsorption of Ethacridine lactate from aqueous solution.

FTIR is a universal valuable analytical tool widely used for materials qualitative evaluation and characterization. For the current research work, the transmittance mode was applied to highlight the specific chemical groups. Figure 4 exhibits the overlay of FTIR examination before and after adsorption. The alginate existence is defined by the specific hydroxyl band at 3200 cm^−1^ [36], vibration stretching of –CH at 2920 cm^−1^ [37,38] and the stretching of carboxylate (–COO) ions at 1600–1400 cm^−1^ [39,40] followed by C–O in the range of 1100 cm^−1^ and 900 cm^−1^ [35]. A –CH_2_ bending vibration close to 1000 cm^−1^ can also be emphasized. Bands of 1630 cm^−1^ and 1540 cm^−1^ can be attributed to amide I and amide II, while between approximatively 1300 cm^−1^ and 1200 cm^−1^, stretching for amide III (proteins) and for asymmetric and symmetric PO^2−^ (phosphorylated proteins and phospholipids), possibly caused by the increased amount of yeast, can be detected [20]. 

Bands in the region of 3500–3000 cm^−1^ from the spectrum acquired after adsorption can be assigned to N–H asymmetric and symmetric stretching vibrations of primary aromatic amine and hydrogen-bonded N–H stretching vibrations of Ethacridine lactate, which confirms its presence in the biosorbent matrix [41]. In conjunction, the signals observed at 1700 cm^−1^, at 1630 cm^−1^ to C=O, C=N stretching vibrations of the lactate anion and acridine ring respectively and those in the region between 850 cm^−1^ and 1100 cm^−1^ corresponding C–H band [42] offer additional indication of the incorporation of ethacridine in the adsorbent. 

**Figure 5:**
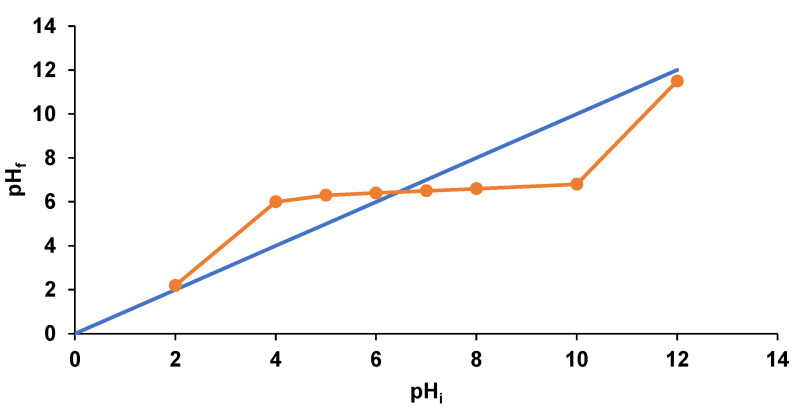
Point of zero charge of the synthesized biosorbent.

With respect of the point of zero charge, establishing its value gives information about the EL biosorption behavior. When the process is conducted at pH values below the pH_PZC_, the biosorbent is positively charged and interactions with the negative charges of EL occur easily insuring a good biosorption. On the other hand, at pH superior to pH_PZC_, the negative charges of the adsorbent material are attracted by the drug positive charges. From Figure 5, it can be remarked that the biosorbent prepared by encapsulation of residual biomass of *Saccharomyces pastorianus* on calcium alginate has a pH_PZC_ value of 6.4. This is close to that found by de Rossi et al. (2020) [17], who developed beads from residual biomass of *Saccharomyces cerevisiae* immobilized on calcium alginate and use them to adsorb metals (lead, chromium, cadmium) from water. The pH_PZC_ was 7, indicating a neutral character of the synthesized material. 

### 3.3. Influence of Working Parameters (pH, Biosorbent Dosage, EL Initial Concentration and Temperature) on the Biosorption Process 

The biosorbent beads were obtained using 2% sodium alginate solution and 5% residual biomass (d.w.). Biomass is found in the prepared solution in an amount 2.5 times higher than sodium alginate. Moreover, the amount of biomass is reported as dry biomass which hydrated during the biosorbent preparation process (cells usually contain up to 76% of water [43], further increasing the cells proportion in the suspension (up to almost 16%, eight times higher than sodium alginate).

Calcium alginate is formed through cross-linking, essential for sodium alginate to build a 3D structure, but also decreasing the biosorption capacity because some sorption sites on sodium alginate are consumed in the reaction [44]. 

Taking into account the much higher quantity of biomass and the fact that alginate has limited sites available for adsorption, the experiments analyzed the biosorbent as a whole. The obtained results regarding the effect of the process influencing parameters are detailed bellow.

#### 3.3.1. Influence of pH

Due to its considerable impact on the biosorption, the initial pH of the EL solution was the first parameter studied in the process of establishing the appropriate conditions for a good retention of the target drug on the prepared adsorbent material. Seven different values (2, 4, 5, 6, 7, 8, 10) were tested with EL solutions having initial concentration of 60 mg/L putted in contact with 2 g/L biosorbent at room temperature. 

**Figure 6:**
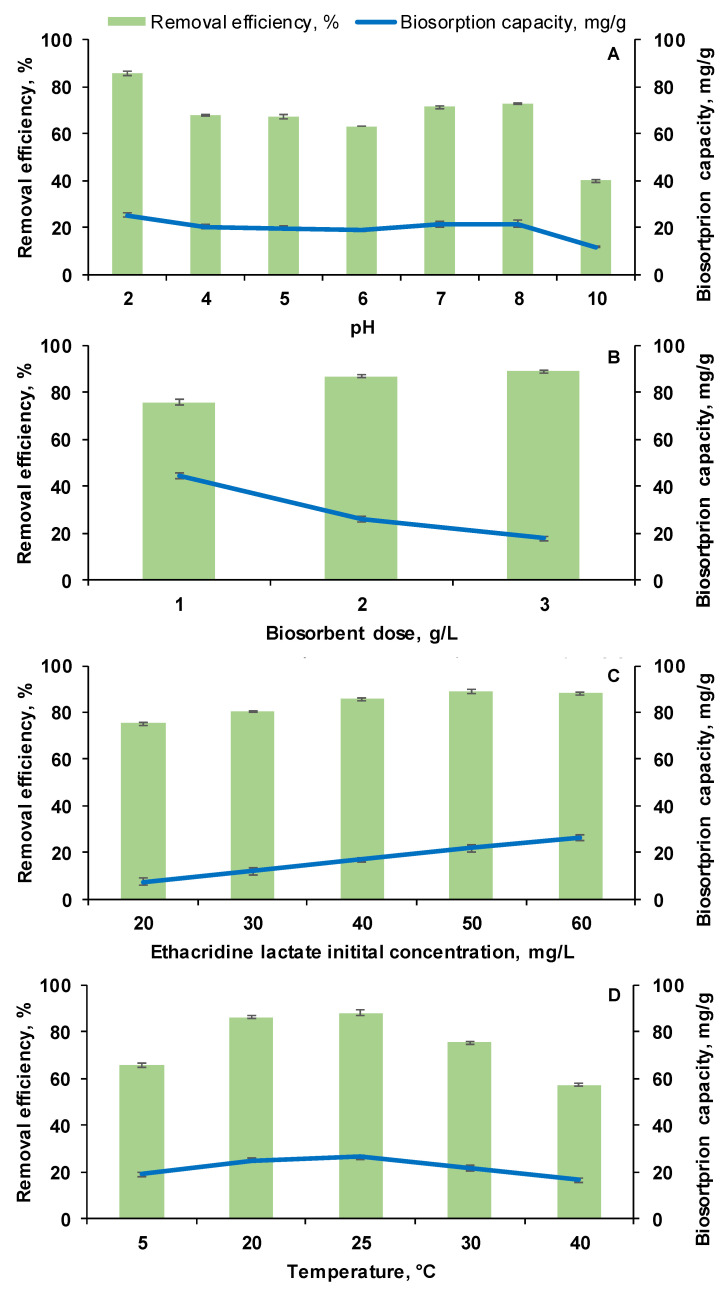
Effect of working conditions on EL removal efficiency and on the biosorption capacity (**A**): influence of pH (EL solution volume: 10 mL; EL initial concentration: 60 mg/L; biosorbent dose: 2 g/L; room temperature); (**B**): influence of biosorbent dose (EL solution volume: 20 mL; EL initial concentration: 60 mg/L; pH: 2; room temperature); (**C**): influence of EL initial concentration (EL solution volume: 30 mL; biosorbent dose: 2 g/L; pH: 2; room temperature); (**D**): influence of temperature (EL solution volume: 10 mL; EL initial concentration: 60 mg/L; biosorbent dose: 2 g/L; pH: 2).

According to data presented in Figure 6A, the higher removal efficiency (87.01 ± 0.67%) of EL from the aqueous solution was obtained at pH 2. Between pH 4 and 8, the removal efficiency was in the range of 68.40 ± 0.45% (pH 4) and 73.50 ± 0.31% (pH 8). The lowest retention was registered at pH 10 at which the removal efficiency reached a value of only 41.01 ± 0.77%. The biosorption capacity followed the same trend, its best value (26.50 ± 0.85 mg/g) being observed in acid media and the lowest in strong alkaline environment (10.80 ± 0.45 mg/g).

The good retention observed at pH 2 can be explained by the fact that Ethacridine lactate is a molecule able to dissociate in aqueous solutions and hence, at acidic pH, it is adsorbed by the biosorbent positively charged surface. Moreover, the biosorbent contains phospholipids from yeast. These amphiphilic molecules which, in aqueous solutions, have the hydrophilic heads exposed to the liquid and the tails directed into the membrane which ensure a hydrophilic environment on the biosorbent surface which is favorable to Ethacridine lactate biosorption due to cation exchange process. The fact that dried biomass used for obtaining the biosorbent hydrates during the preparation process [43] sustains also the hypothesis of the hydrophilic environment. 

Similar explanations are given also by Okada et al. (1987) [45] and by Talman et al. (2015) [2]. Okada et al. (1987) [45] studied the adsorption of various drugs on microcrystalline cellulose. They explain that Ethacridine lactate, a salt of basic ethacridine (pK_a1_ = 11.6) and lactic acid (pK_a2_ = 3.86), can exist in two forms: a diprotic form, caused by the protonation of 6-NH_2_ group of the acridine ring, and a monoprotic form. Therefore, it dissociates in aqueous solutions and hence, at acidic pH, it is adsorbed by the biosorbent positively charged surface. Talman et al. (2015) [2] focused also on the adsorption of EL from aqueous solutions. In their case, when the procedure was carried out on activated carbon, better results were obtained in alkaline media, while the pH did not significantly influence the retention of the drug on bentonite. They arrived at the conclusion that the adsorption of EL can be attributed to an ion-exchange process and to electrostatic attraction between the EL and adsorbents.

As consequence of the above related aspects, the following experiments were realized at pH 2.

#### 3.3.2. Influence of Biosorbent Dosage 

Biosorbent dose is recognized as being other important operational parameter for the adsorption processes. Three levels of biosorbent were considered for our experimental setup with EL initial concentration of 60 mg/L, at pH 2 and room temperature. As expected, the removal efficiency increased with the increase of the adsorbent material amount from 74.01 ± 1.12% (1 g/L) to 86.09 ± 0.55% (2 g/L) due to the availability of more adsorption sites. From Figure 6B, it can be seen that an augmentation of the biosorbent dose from 2 g/L to 3 g/L amplifies the removal efficiency with only 2% (up to 88.01 ± 0.61%). The analysis of the biosorption capacity evolution reveals the higher value (43.50 ± 1.25 mg/L) at the lowest adsorbent dose and smaller but rather close values for the other two doses. 

Our data are similar with those reported by Adel Niaei and Rostamizadeh (2020) [46], who performed the adsorption of metformin on zeolites and showed that the removal percentage improves from 61.1% to more than 95% by increasing the amount of adsorbent from 0.5 g/L to 1.1 g/L. An adsorbent dosage of 2 g/L was found to be adequate also for the retention of metronidazole from wastewater [47]. Based on these aspects, the middle biosorbent dose (2 g/L) was considered a good compromise between the consumption of adsorbent material and the removal of EL from tested solutions. 

#### 3.3.3. Influence of EL Initial Concentration 

The Ethacridine lactate initial concentration (ranging from 20 mg/L to 60 mg/L) was the next factor examined. The EL solution pH was adjusted at 2, the biosorbent dose was 2 g/L and the working temperature was that of the laboratory ambient. 

As illustrated in Figure 6C, the removal efficiency increased from 76.27 ± 0.610% (for drug initial concentration of 20 mg/L) to 88.06 ± 0.98% (for EL initial concentration of 50 mg/L). A slight decrease to 88.01 ± 0.10%, at 60 mg/L of target compound initial concentration, can be remarked showing that, in the studied conditions, the biosorbent is about to reach a plateau. Nonetheless, the biosorption capacity does not pursue the same direction since it raises from 5.49 ± 1.32 mg/g (at EL initial concentration of 20 mg/L) to 26.03 ± 1.05 mg/g (at EL initial concentration of 60 mg/L). The made observations suggest that the biosorption process is affected by the initial pollutant concentration. As the quantity of the used biosorbent is fixed, the available sites for the adsorption are limited. When the contaminant concentration attains a certain value, the adsorption sites will be all occupied and the equilibrium will be achieved.

#### 3.3.4. Influence of Temperature 

Volumes of 10 mL of EL with an initial concentration of 60 mg/L with pH adjusted at 2 were placed in laboratory flasks. The amount of the newly synthesized biosorbent was of 2 g/L. The EL biosorption was conducted at five different temperatures: 5 °C, 20 °C, 25 °C, 30 °C and 40 °C. The effect of this parameter is illustrated on Figure 6D. It appears that the working temperature has a significant impact on EL biosorption. The removal efficiency increases with temperature augmentation from 5 °C to 25 °C where it reaches a maximum of 88.01 ± 1.01%. After that, it decreases to 56.08 ± 0.60% when the temperature is set at 40 °C. The same tendency, with a maximum of 26.06 ± 1.17 mg/g at 25 °C and the lowest value of only 16.00 ± 1.16 mg/g at 40 °C, can be observed for the biosorption capacity. The fact that the increase in temperature is rather unfavorable can be explained by the weaker adsorption forces between the active sites of the biosorbent and the Ethacridine lactate molecules [48]. As consequence, the ambient temperature of 25 °C can be considered as appropriate for conducting the biosorption. 

### 3.4. Biosorption Kinetics

The kinetic experiments were conducted at pH 2 and at a temperature of 25 °C. The biosorbent dosage was set at 2 g/L and the concentrations of EL aqueous solutions varied between 20 mg/L and 60 mg/L. Since, in the biosorption processes, the reaction pathways are influenced by the contact time, samples were collected at specific times and analyzed in order to determine the residual pollutant concentrations.

Pseudo-first-order, pseudo-second-order and intraparticle diffusion models were tested for establishing the appropriate behavior of EL biosorption kinetic. Pseudo-first-order model is constantly used to fit the kinetic data and to calculate the equilibrium adsorption amount and to define how fast the equilibrium is reached while pseudo-second-order model correlates the adsorption with the active sites existing on the adsorbent material [49]. Intraparticle diffusion model assumes that the adsorption rate is controlled by one of the three steps of the process: film diffusion, adsorbate diffusion into the pores and surface adsorption [50].

Among the kinetic models tested, the pseudo-second-order Equation (3) and intraparticle diffusion Equation (4) were the most adequate to describe the EL biosorption.

(3) $$\frac{t}{q_{t}}=\frac{1}{k_{2}\cdot q_{e}^{2}}+\frac{t}{q_{e}}$$

(4) $$q_{t}=k_{i}\cdot t^{0.5}+C$$

where *t* is the reaction time (min); *q_t_* is the biosorption capacity of EL at time *t* (mg/g); *q_e_* is the biosorption capacity of EL at equilibrium (mg/g); *k*_2_ is the biosorption rate constant for pseudo-second-order kinetic model (1/min); *k_i_* is biosorption rate constant for intraparticle diffusion kinetic model (mg/(g min^0.5^) and *C* is the constant associated with boundary layer thickness (mg/g)).

**Figure 7:**
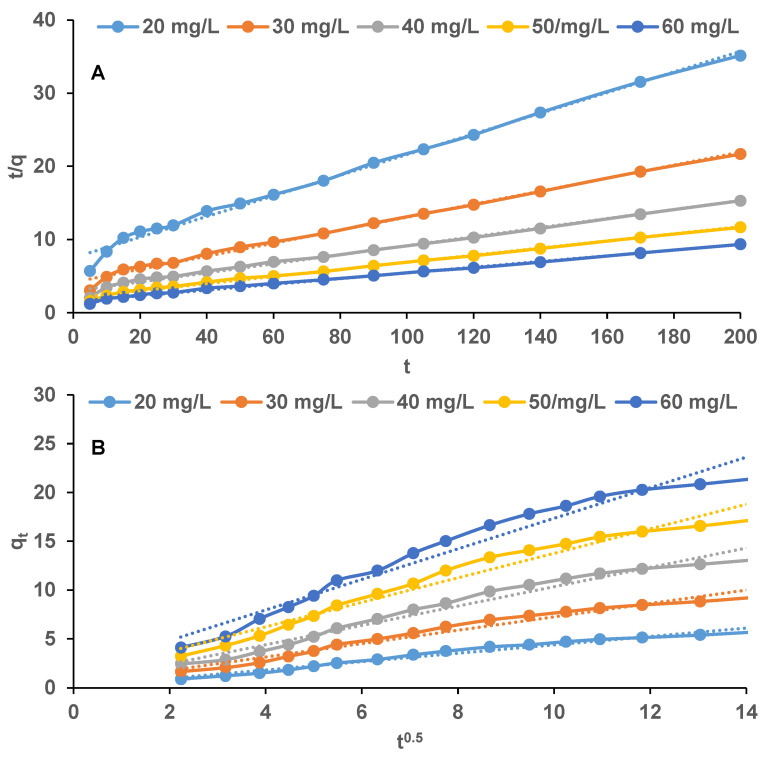
Pseudo-second-order (**A**) and intraparticle diffusion (**B**) kinetic models for the biosorption of EL on the synthesized biosorbent.

The kinetic parameters can be deduced from time-concentration data derived from the interactions occurring between the target molecule and the adsorbent material (Figure 7) and are summarized in Table 1. 

The pseudo-second-order model describes best (R^2^ > 0.99 in all cases) the adsorption of EL on the biosorbent obtained by encapsulating the residual biomass of *Saccharomyces pastorianus* on calcium alginate sustaining that the chemical adsorption contributes to EL biosorption. The theoretical *q_e_* values 5.69 mg/g (for EL initial concentration of 20 mg/L) and 21.39 mg/g (for EL initial concentration of 60 mg/g) were comparable to the experimental ones. On the other part, the values of boundary layer constant *C* of the intraparticle diffusion model, which are superior to zero, indicate the fact that intraparticle diffusion was not the only rate-limiting step and that there are other different mechanisms occurring during the process.

Similar findings are disclosed by Yadav et al. (2021) [51], who synthesized nanocomposite beads using iron oxide, activated charcoal, β-ciclodextrin and sodium alginate, and used them to adsorb, among others, different drugs. According to their results, there were at least two processes involved in drugs adsorption: an external diffusion of the molecules on the adsorbent surface and an intraparticle diffusion of the adsorbate into internal pores of the adsorbent material. They also highlight that the pseudo-second-order kinetic is predominant showing that the chemical adsorption is a rate-limiting step of the process. The pseudo-second-order and intraparticle diffusion kinetics acceptably described also the adsorption of other drugs on various adsorbents such as ibuprofen and naproxen on activated carbon obtained from murumuru waste by chemical treatment with zinc chloride [52], diclofenac and carbamazepine on silica-based porous materials [53], sulfomethoxazole, carbamazepine, ketoprofen, naproxen, diclofenac and ibuprofen on phosphorized microporous carbonous material [54], etc. 

### 3.5. Equilibrium Isotherms 

Different equilibrium isotherms, i.e., Langmuir, Freundlich, Dubinin–Radushkevich and Temkin were employed to validate the uptake behavior of Ethacridine lactate by the biosorbent obtained by encapsulation of *Saccharomyces pastorianus* residual biomass on calcium alginate. Langmuir model specifies that the adsorption does not occur beyond the monolayer coating, that the adsorption sites are equivalent and the adsorbent surface is uniform and that the there are no interactions between the adsorbed molecules [55]. Freundlich isotherm considers adsorption to be a multi-layer process in which the amount of adsorbate increases gradually [56]. The Dubinin–Radushkevich model implies that the adsorption process is related to pores volume filling contrasting to layer-by-layer adsorption on pore walls [57]. Temkin isotherm model assumes that the process heat is influenced by the adsorption of target molecules on the adsorbent [58].

Of the practiced isotherm models, Freundlich Equation (5) and Dubinin–Radushkevich Equation (6) were those that were in good agreement with the experimental data.

(5) $$\log q_{e}=\log K_{F}+\frac{1}{n}\cdot \log C_{e}$$

(6) $$\ln q_{e}=\ln q_{m}-\beta \cdot \varepsilon^{2}$$

in which *C_e_* is the equilibrium concentration (mg/L); *q_e_* is the equilibrium biosorption capacity (mg/g); *n* and *K_F_* (mg/g) are the Freundlich constants; *q_m_* is the maximum biosorption capacity (mg/g); *β* (mol^2^/kJ^2^) and *ε* are the Dubinin–Radushkevich constants with the mean free energy $E=\frac{1}{\sqrt{2\cdot \beta }}$ (kJ/mol) and the Polanyi potential.

$$\varepsilon =R\cdot T\cdot \ln\left(1+\frac{1}{C_{e}}\right)$$

where *R* and *T* are the gas constant (J/(mol K)) and absolute temperature (K) respectively.

**Figure 8:**
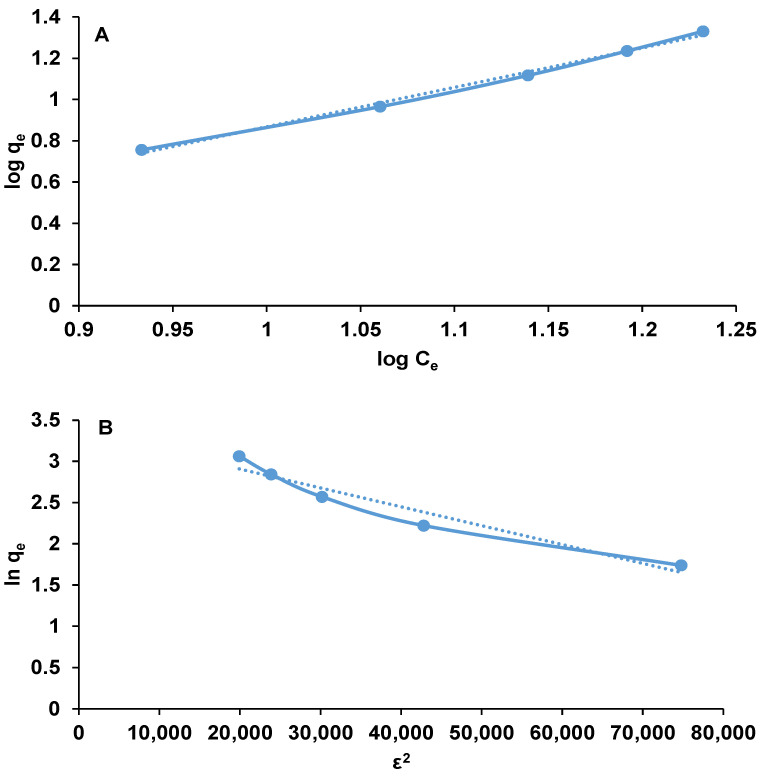
Freundlich (**A**) and Dubinin–Radushkevich (**B**) equilibrium isotherms for the biosorption of EL on the synthesized biosorbent.

The parameters of the Freundlich and Dubinin–Radushkevich isotherm were calculated from the slope and intercept of the linear plots (Figure 8) and are given in Table 2. 

In Freundlich model, the constant *K_F_* is affected by the adsorption capacity while the other constant *n* fluctuates with the adsorbent heterogeneity its value revealing whether the adsorption is chemical (*n* < 1), physical (*n* > 1) or linear (*n* = 1). In the case of the present research, the *n* constant has a low value of 0.5232 which shows the favorable chemical EL adsorption on the obtained biosorbent. The high correlation coefficient of Freundlich model (R^2^ > 0.9942) suggest that the adsorption occurred on a heterogeneous surface and that the EL molecules interacts with each other. 

The Dubinin–Radushkevich isotherm states if the adsorption interactions are chemical or physical. When the calculated mean sorption energy *E* is lower than 8 kJ/mol, the adsorption is a physical one; when *E* is ranging from 8 kJ/mol and 16 kJ/mol, an ion-exchange mechanism is considered as controlling the process; when *E* is higher than 16 kJ/mol (which is the situation of this study with *E* = 147.442 kJ/mol), the adsorption is a chemical one [59]. This outcome is coherent with the assumptions made earlier for Freundlich model.

Data published by Gholamiyan et al. (2020) [60] or by Kim and Kim (2019) [61] reveal that the adsorption of erythromycin on magnetic activated carbon or of paclitaxel on Sylopute are also described by the above-mentioned models.

## 4. Conclusions

The biosorbent based on *Saccharomyces pastorianus* residual biomass encapsulated in a calcium alginate matrix was synthesized and characterized by SEM and FTIR techniques. The obtained beads have an irregular and elongated shape with 1.89 mm ± 0.13 mm in size. SEM analysis shows a homogeneous structure with uniform pore distribution and rolling tendencies observed before EL biosorption while a smoother surface can be noticed after biosorption. These changes in beads morphologies along with the results of the FTIR analysis sustain the fact that the EL was retained by the biosorbent. For a better understanding of EL biosorption behavior, the point of zero charge was also determined. Its value was of 6.4.

Systematic studies of biosorption parameters were performed and analyzed. The pH of the initial solution and temperature played an important role on the EL removal. According to recorded data, the best removal efficiency for EL of over 85% was obtained at acidic pH 2 and 25 °C for 50 mg/L initial concentration and 2 g/L biosorbent dose. The pseudo-second-order and intraparticle diffusion kinetics describe the biosorption process. The maximum calculated biosorption capacity was 21.39 mg/g similar to that recorded experimentally. The equilibrium biosorption data were a good fit for the Freundlich and Dubinin–Radushkevich isotherms with correlation coefficients higher than 0.99, respectively 0.93.

Finally, these findings sustain the idea that the *Saccharomyces pastorianus* residual biomass–calcium alginate system is a promising material for the biosorption of Ethacridine lactate from aqueous media.

## Figures and Tables

**Table 1:** Biosorption kinetic parameters of EL on synthesized biosorbent.

Kinetic Model	EL Initial Concentration, Mg/L	Kinetic Parameters				Correlation Coefficient, R^2^
		*Q_e_*	*K*_2_	*K_i_*	*C*	
Pseudo-second-order	20	5.6932	0.0031	-	-	0.9917
	30	9.2236	0.0062	-	-	0.9927
	40	13.0694	0.0086	-	-	0.9906
	50	17.1651	0.0144	-	-	0.9944
	60	21.3909	0.0194	-	-	0.9951
Intraparticle diffusion	20	-	-	0.4321	0.0587	0.9755
	30	-	-	0.6879	0.3664	0.9696
	40	-	-	0.9889	0.4344	0.9703
	50	-	-	1.2558	1.1923	0.9651
	60	-	-	1.5669	1.6801	0.9618

**Table 2:** Freundlich and Dubinin–Raduschkevich isotherm models of EL biosorption process on *Saccharomyces pastorianus*/calcium alginate beads.

Parameter	Freundlich Model	Dubinin–Radushkevich Model
*n*	0.5232	-
*K_F_* (mg/g)	2.8394	-
*q_m_* (mg/g)	-	28.8765
*β* (mol^2^/kJ^2^)	-	0.000023
*E* (kJ/mol)	-	147.4420
R^2^	0.9942	0.9369

## Data Availability

All data produced in this study are presented in this paper.
